# Multicenter evaluation of crystal violet decolorization assay (CVDA) for rapid detection of isoniazid and rifampicin resistance in *Mycobacterium tuberculosis*

**DOI:** 10.1038/srep39050

**Published:** 2016-12-16

**Authors:** Ahmet Yilmaz Coban, Ahmet Ugur Akbal, Can Bicmen, Ali Albay, Ali Korhan Sig, Meltem Uzun, Deniz Sertel Selale, Nuri Ozkutuk, Suheyla Surucuoglu, Nurhan Albayrak, Nilay Ucarman, Aydan Ozkutuk, Nuran Esen, Ismail Ceyhan, Mustafa Ozyurt, Bayhan Bektore, Gonul Aslan, Nuran Delialioğlu, Alpaslan Alp

**Affiliations:** 1 Ondokuz Mayis University Medical School Department of Medical Microbiology, Samsun, Turkey; 2 Dr. Suat Seren Chest Diseases and Chest Surgery Training and Research Hospital, Medical Microbiology Laboratory, Izmir, Turkey; 3 Gulhane Military Medical Academy, Department of Medical Microbiology, Ankara, Turkey; 4 Istanbul University Istanbul Medical School Department of Medical Microbiology, Istanbul, Turkey; 5 Celal Bayar University Medical School Department of Medical Microbiology, Manisa, Turkey; 6 Public Health Agency of Turkey Tuberculosis Reference Laboratory, Ankara, Turkey; 7 Dokuz Eylul University Medical School Department of Medical Microbiology, Izmir, Turkey; 8 Atatürk Chest Diseases and Chest Surgery Training and Research Hospital Medical Microbiology Laboratory, Ankara, Turkey; 9 Haydarpasa Military Medical Academy, Department of Medical Microbiology, Istanbul, Turkey; 10 Mersin University Medical School Department of Medical Microbiology, Mersin, Turkey; 11 Hacettepe University Medical School Department of Medical Microbiology, Ankara, Turkey

## Abstract

The aim of this multicenter study was to evaluate the performance of the crystal violet decolorization assay (CVDA) for detection of multidrug resistant tuberculosis (MDR-TB). This study was performed in 11 centers in two phases. A total of 156 isolates were tested for INH and RIF resistance. In the phase I, 106 clinical isolates were tested in the Center 1–7. In the phase 2, 156 clinical isolates were tested in the center 1–6, center 8–11. Eighty six of 156 tested isolates were the same in phase I. Agreements were 96.2–96.8% for INH and 98.1–98.7% for RIF in the phase I-II, respectively. Mean time to obtain the results in the phase I was 14.3 ± 5.4 days. In the phase II, mean time to obtain the results was 11.6 ± 3.5 days. Test results were obtained within 14days for 62.3% (66/106) of isolates in the phase I and 81.4% (127/156) of isolates in the phase II. In conclusion, CVDA is rapid, reliable, inexpensive, and easy to perform for rapid detection of MDR-TB isolates. In addition, it could be adapted for drug susceptibility testing with all drugs both in developed and developing countries.

Tuberculosis is an important health problem for worldwide, especially, in developing countries^1^. The most important step in preventing the spread of tuberculosis is diagnosis and rapid detection of drug resistance^2^. Recently, increasing of multidrug resistant tuberculosis (MDR-TB) underscores the need of rapid, accurate and reliable methods for detection of drugs resistance especially isoniazid (INH) and rifampicin (RIF)^2^,^3^.

MDR-TB, defined as resistance to at least INH and RIF from the first line drugs, complicates effective TB control program. The rapid detection of MDR-TB is important issue for reducing the transmission of the disease^4^. There are standardized methods for drug susceptibility testing at hospital setting. Proportion method performed on Middlebrook 7H10–11 agar and Löwenstein Jensen (LJ) medium, absolute concentration and resistance ratio methods are conventional methods. Preparation of media contain antibiotics used in these methods is a labor intense process. Furthermore, obtaining the test results by conventional method stakes 3–6 weeks^5^,^6^,^7^. Therefore, these methods are not preferred in routine laboratory works, nowadays. Commercial systems easily performed and yield test results in a shorter time period have been developed. These systems include automatized commercial systems BACTEC MGIT 960 (Becton Dickinson, ABD), Versa Trek (Thermofischer, ABD) and TK medium (Salubris, Turkey). BACTEC MGIT 960, recommended by CLSI for drug susceptibility testing, is most commonly used^5^,^6^,^7^,^8^. Although the results are obtained faster, they have important disadvantage including high coast and the need of technical equipment^7^,^8^. Therefore, these systems cannot be used in countries with low socioeconomics^9^,^10^.

Tuberculosis is mainly seen in developing countries. Increased MDR-TB cases in these countries made cheap, reliable, rapid and easily performed susceptibility testing methods mandatory. Phenotypic methods developed recently seems to be both rapidness and cost. These methods are colorimetric methods (resazurin microplate method or resazurin tube test^11^, malachite green decolorization assay^12^,^13^, nitrate reductase test)^10^, gradient diffusion method (E-test)^14^, microscopic observation drug susceptibility assay^15^, and thin-layer agar method^16^. In comparing these new methods with each other, they have advantage and disadvantage in performing and evaluation stages. Colorimetric susceptibility test methods are preferred for rapid detection of resistance; because they are rapid, reliable, and inexpensive and can be performed and interpreted easily as well^10^. The crystal violet decolorization assay (CVDA) is one of the colorimetric susceptibility test methods. Crystal violet is triphenylmethane dye that is antimicrobial, toxic to mammalian cells, and mutagenic. In addition mycobacterium is resistant to crystal violet. Mycobacterial resistance to crystal violet could be due to the reduction of the dyes and the sequestering of the dyes in the lipid fraction of cells^17^.

In this multi-central study, a novel colorimetric CVDA were tested. Evaluation and application of the test is extremely easy and it can be considered that it can be used for rapid diagnosis of MDR-TB in laboratories with limited resources.

## Materials and Methods

### Study centers

This study was performed in 11 centers including; Center 1 (C1); Ondokuz Mayis University Medical School Department of Medical Microbiology, Center 2 (C2); Istanbul University Istanbul Medical School Department of Medical Microbiology, Center 3 (C3); Dr. Suat Seren Chest Disease and Chest Surgery Training and Research Hospital Medical Microbiology Laboratory, Center 4 (C4); Gulhane Military Medical Academy Department of Medical Microbiology, Center 5 (C5); Celal Bayar University Medical School Department of Medical Microbiology, Center 6 (C6); Public Health Agency of Turkey Tuberculosis Reference Laboratory, Center 7 (C7); Dokuz Eylul University Medical School Department of Medical Microbiology, Center 8 (C8); Atatürk Chest Disease and Chest Surgery Training and Research Hospital Medical Microbiology Laboratory, Center 9 (C9); Haydarpasa Gulhane Military Medical Academy Department of Medical Microbiology, Center 10 (C10); Mersin University Medical School Department of Medical Microbiology and Center 11 (C11); Hacettepe University Medical School Department of Medical Microbiology.

The study was performed in two phases.

### Bacterial strains

In this study, a total of 156 isolates were tested for INH and RIF. In the phase I (106 clinical isolates), 15, 17, 19, 13, 10, 12 and 20 isolates were tested in the C1, C2, C3, C4, C5, C6 and C7, respectively. In the phase 2 (156 clinical isolates), 15, 20, 20, 13, 10, 14, 20, 20, 18 and 6 isolates were tested in the C1–6, C8–11, respectively.

### Preparation of 7H9S broth for the CVDA test

#### Phase I

Seven centers (C1–7) were participated the phase I and these centers tested 106 isolates. In this phase, all isolates were tested to INH (0.25 mg/L) and RIF (0.5 mg/L) for CVDA. Crystal violet stock solution and three test tubes including INH, RIF and drug-free growth control tube were prepared at the C1 and were sent to each center in a cold chain condition. In addition, test procedure was also sent to each center to provide harmonization.

#### Phase 2

All bacterial isolates from the centers except C7 were sent to the C1 and 156 clinical isolates were tested for CVDA in the C1. Eighty six of 156 tested isolates were the same in phase I. The test procedure was the same in the phase I except INH concentration. In this phase study INH was tested in 0.125 mg/L concentration.

### Preparation of CV

CV stock solution was prepared in sterile distilled water as 25 mg/L and sterilized by filtration. Stock solution was kept on +4 °C until used.

### Preparation of bacterial inoculums

The colonies freshly grown on LJ medium were put into screw-cap tubes with 4–5 ml sterile serum physiologic and glass beads and vortexed for 30 s or 1 min. After vortex, all tubes were kept in a vertical position for 1 hour at room temperature to sediment large particles and aerosols. The bacterial suspensions were adjusted to McFarland no 1 standard.

### Performing the test

#### Phase I

**Figure 1:**
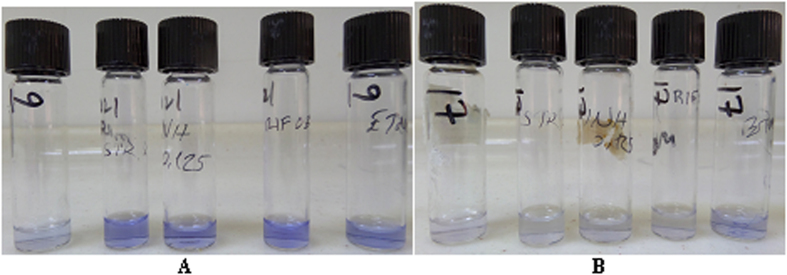
(**A**) Susceptible to INH, RIF, STM, EMB, (**B**) Resistant to INH, RIF, STM, EMB.

Seven centers, participated to this phase inoculated 50 μl bacterial inoculum into each tube. Subsequently, tubes were incubated at 37 °C. At the end of 7^th^ day of incubation, 100 μl of CV solution were added into the each tube and kept on incubation at 37 °C. After that tubes were checked every day until decolorization was seen in the growth control tube. Results were evaluated when decolorization was seen in the growth control tube. If decolorization was seen in the drug containing tube, the bacterium was reported as resistant (Fig. 1).

#### Phase II

In this phase, all isolates from all centers except C7 were collected in C1 and tested again. The concentration of 0.25 mg/L for INH was considered as high level therefore; it was tested in the concentration of 0.125 mg/L in that phase. Test procedure was similar with the phase I.

## Results

Center 1–6 participated to both phases I and II of the study whereas C7 participated only to the phase I and C8–11 participated only to the phase II. Tested bacteria in both phases of the study were presented in Table 1. A total of 106 isolates including 41 MDR, 4 INH mono resistant, 2 RIF mono resistant and 59 susceptible isolates were tested in the phase I whereas a total of 156 isolates including 56 MDR, 15 INH mono resistant, 4 RIF mono resistant and 81 susceptible isolates were tested in the phase II (Table 1).

The concentration of INH was 0.25 mg/L in the phase I but it was decreased to 0.125 mg/L in the phase II. As a result of this change, one isolate from C2, determined as resistant by reference method was determined susceptible in the phase I whereas it was determined resistant in the phase II. One isolate from C1 was resistant according to reference method whereas it was susceptible in the phase II but it was resistant in the phase I. Another isolate from C6 was susceptible by reference method but it was resistant in both phase I and II.

Results of the study, performed in two stages, were evaluated in comparing with standard method for each stage. BACTEC MGIT 960 system was used as gold standard in the study. Obtained results both in stage I and II were compared with gold standard for the calculation of concordance, sensitivity, specificity, positive and negative predictive values of the method.

Excellent agreements were observed, and they were 96.2–96.8% for INH and 98.1–98.7% for RIF in the phase I-II, respectively. In the phase I and II, sensitivity, specificity, positive predictive value (PPV) and negative predictive value (NPV) for INH and RIF were 97.7–97.1/100–96.8, 95.2–96.5/96.9–100, 93.3–95.7/95.3–100 and 98.3–97.7/100–97.9, respectively (Table 2).

In addition, validation of the critical antibiotic concentration used in CVDA test were performed and determined as 0.125 and 0.5 mg/L for INH and RIF, respectively.

**Figure 2:**
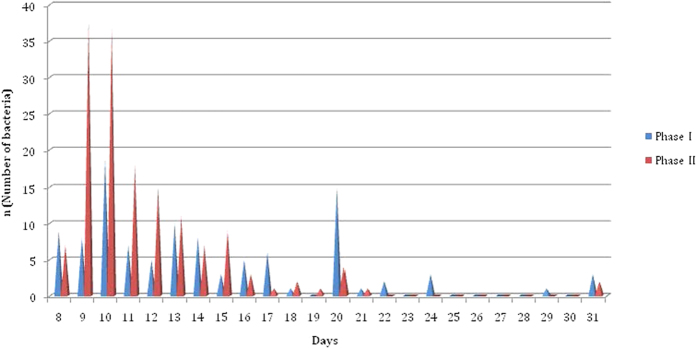
Distribution of time to detection of the results in the Phase I and Phase II.

Distribution of time to detection of the results in the phase I and II were summarized in the Fig. 2. Mean time to obtain the results of susceptible and resistant isolates in the phase I was 13.1 ± 4.5 and 15.8 ± 6.1 days, respectively. It was 14.3 ± 5.4 days for all isolates. In the phase II, mean time to obtain the results was 10.6 ± 2.2 for susceptible isolates whereas it was 12.6 ± 4.3 days. It was 11.6 ± 3.5 days for all isolates. Test results were obtained within 14 days for 62.3% (66/106) of isolates in the phase I and 81.4% (127/156) of isolates in the phase II (Fig. 2).

## Discussion

The most important step for tuberculosis control program is early detection of TB in order to prevent the spread within the community. For this reason, rapid, inexpensive and reliable susceptibility test methods are needed. However, the timely determination of resistance profile remains a major problem due to the slow growth of *M. tuberculosis*^18^.

CVDA was developed by Coban^19^ in 2014. In his study, sensitivity and specificity were 92.5% and 96.4%for INH and were 88.8% and 100% for RIF. Coban *et al*.^20^ evaluated CVDA for MIC detection of primary anti-tuberculosis drugs. They found that the agreements were 98.1, 100, 96.2 and 98.1 for INH, RIF, STM and EMB, respectively.

This study is the first multicenter study that evaluated the CVDA. Excellent agreements were observed for rapid detection of MDR-TB. The agreements for the phase I and II were 96.2 and 96.8% for INH and 98.1 and 98.7% for RIF, respectively. On the other hand, time to obtain the results for the phase I and II were 14.3 ± 5.4 day and 11.6 ± 3.5 day, respectively.

In the proportion method, inoculum with McFarland no 0.5–1 turbidity is diluted as 1:100 before the inoculation into 100 μl media^9^. In resazurin microtitre assay test, bacterial inoculums with McFarland no 1 turbidity is diluted as 1:20 before inoculation and inoculated as 100 μl into 100 μl media containing drug (final dilution 1:40)^21^. However, 50 μl of bacterial inoculum with McFarland no 1 turbidity is inoculated into 1 ml of medium containing drug in CVDA method (final dilution 1:20). Therefore, higher bacterial concentration used in CVDA method can increase the detection probability of resistant mutant bacteria.

Crystal violet can be reductively decolorized. This decolorization occurs during *M. tuberculosis* metabolism^17^,^22^. Therefore, time to obtain the results depends on the metabolic activity of the bacteria. It can be considered that the longer time (31 days) to obtain the results for some isolates in the study can be in consequence of slow metabolic activity of these bacteria. Nevertheless, this should be elucidated by further investigations. Another advantage of the test is the ability to monitor the test for longer time until decolorization occurs. Results of 25 isolates were obtained at the 20^th^ day and later in the phase I of the study whereas it was only for 7 isolates in the phase II. It may be due to using fresh grown bacteria and some disruptions (the majority of the results were obtained at the 20^th^ day in one center) in periodic evaluation of the test.

The resazurin microplate method, the nitrate reductase assay, MTT, XTT and the malachite green decolorization assay have been developed for the drug susceptibility testing. The nitrate reductase assay requires three different compounds for obtaining Griess reagent used in the testing. After the preparation oh this reagent, it should be used freshly in the test. Although the preparation and standardization of LJ medium is very difficult, the test is performed commonly on this medium^6^,^19^. Resazurin powder increases cost burden of the resazurin microplate method. There is less information in the literature regarding the XTT and MTT. In addition, MTT requires further extraction to dissolve the formazon, but not XTT^23^. However, inexpensive crystal violet dye can be easily obtained because it is commonly used for Gram staining in hospital setting laboratories^19^.

In summary, results of this multicenter study show that CVDA is rapid, reliable, inexpensive, and easy to perform for rapid detection of MDR-TB isolates. In addition, it could be adapted for drug susceptibility testing with all drugs both in developed and developing countries.

## Additional Information

**How to cite this article**: Coban, A. Y. *et al*. Multicenter evaluation of crystal violet decolorization assay (CVDA) for rapid detection of isoniazid and rifampicin resistance in *Mycobacterium tuberculosis. Sci. Rep.* **6**, 39050; doi: 10.1038/srep39050 (2016).

**Publisher's note:** Springer Nature remains neutral with regard to jurisdictional claims in published maps and institutional affiliations.

## Figures and Tables

**Table 1:** Resistance profiles of clinical isolate tested in 11 centers.

	Isolates	C1	C2	C3	C4	C5	C6	C7	C8	C9	C10	C11	Total
**Phase I**	**MDR**	4	7	8	3	3	8	8	—	—	—	—	41
	**INH-mono R**	3	0	1	0	0	0	0	—	—	—	—	4
	**RIF- mono R**	0	0	0	0	0	0	2	—	—	—	—	2
	**Susceptible**	8	10	10	10	7	4	10	—	—	—	—	59
	**Total**	15	17	19	13	10	12	20	—	—	—	—	106
**Phase II**	**MDR**	4	8	9	3	3	9	—	8	7	1	4	56
	**INH- mono R**	3	2	1	0	0	1	—	2	1	5	0	15
	**RIF- mono R**	0	0	0	0	0	0	—	4	—	—	—	4
	**Susceptible**	8	10	10	10	7	4	—	6	12	12	2	81
	**Total**	15	20	20	13	10	14	—	20	20	18	6	156

C: center; INH: isoniazid; RIF: rifampicin; MDR: multidrug resistant; R: resistant; S: susceptible; mono R: mono resistant.

**Table 2:** Comparison of the CVDA and the reference method results.

Drugs	CVRT	Reference method		Sensitivity (%)	Specificity (%)	PPV (%)	NPV (%)	(%)
		R	S					
**INH**	R	42	1	97.7	95.2	93.3	98.3	96.2
**Phase I**	S	3	60					
**RIF**	R	41	0	100	96.9	95.3	100	98.1
	S	2	63					
**INH**	R	67	2	97.1	96.5	95.7	97.7	96.8
**Phase II**	S	3	84					
**RIF**	R	60	2	96.8	100	100	97.9	98.7
	S	0	94					

INH: isoniazid; RIF: rifampicin; PPV: positive predictive value; NPV: negative predictive value.
